# The Bipolar Interactive Psychoeducation (BIPED) study: trial design and protocol

**DOI:** 10.1186/1471-244X-9-50

**Published:** 2009-08-12

**Authors:** Sharon Simpson, Emma Barnes, Emily Griffiths, Kerry Hood, David Cohen, Nick Craddock, Ian Jones, Daniel J Smith

**Affiliations:** 1 South East Wales Trials Unit, Department of Primary Care and Public Health, 7thfloor Neuadd Merionnydd, School of Medicine, Cardiff University, Heath Park, Cardiff, UK, CF14 4XN; 2 Department of Psychological Medicine, Cardiff University School of Medicine, Monmouth House, Heath Park, Cardiff, UK, CF14 4DW; 3 Health Economics and Policy Research Unit, University of Glamorgan, Pontypridd, UK, CF37 1DL

## Abstract

**Background:**

Bipolar disorders affect between 3–5% of the population and are associated with considerable lifelong impairment. Since much of the morbidity associated with bipolar disorder is caused by recurrent depressive symptoms, which are often only poorly responsive to antidepressants, there is a need to develop alternative, non-pharmacological interventions. Psychoeducational interventions have emerged as promising long-term therapeutic options for bipolar disorder.

**Methods/design:**

The study is an exploratory, individually randomised controlled trial. The intervention known as 'Beating Bipolar' is a psychoeducational programme which is delivered via a novel web-based system. We will recruit 100 patients with a diagnosis of DSM-IV bipolar disorder (including type I and type II) currently in clinical remission. The primary outcome is quality of life. This will be compared for those patients who have participated in the psychoeducational programme with those who received treatment as usual. Quality of life will be assessed immediately following the intervention as well as 10 months after randomisation. Secondary outcomes include current depressive and manic symptoms, number of episodes of depression and mania/hypomania experienced during the follow-up period, global functioning, functional impairment and insight. An assessment of costs and a process evaluation will also be conducted which will explore the feasibility and acceptability of the intervention as well as potential barriers to effectiveness.

**Discussion:**

Bipolar disorder is common, under-recognised and often poorly managed. It is a chronic, life-long, relapsing condition which has an enormous impact on the individual and the economy. This trial will be the first to explore the effectiveness of a novel web-based psychoeducational intervention for patients with bipolar disorder which has potential to be easily rolled out to patients.

**Trial registration:**

Current Controlled Trials ISRCTN81375447

## Background

Bipolar disorder is a severe recurrent disorder of mood and behaviour characterised by episodes of depression and mania or hypomania[1]. A clinical spectrum of bipolar disorder affects between 3–5% of the population and results in considerable lifelong social and occupational impairment [2-4]. Most of the morbidity associated with this disorder is caused by recurrent depressive episodes and chronic, low-grade depressive symptoms, which are present in most patients for at least half of their life-time[5,6].

There is currently considerable uncertainty about how best to treat these chronic depressive symptoms which have an enormous impact on the quality of life and functioning of large numbers of bipolar patients [7]. Much of this uncertainty concerns the use of antidepressants. Several studies suggest that a significant proportion of patients (between 30–50%) will respond poorly to antidepressants and may develop a chronic, cycling course of illness with more frequent depressive episodes, more suicidal behaviour and, in the longer-term, more treatment-resistance [8,9]. As a result, there is an urgent need to develop alternative, non-pharmacological interventions which might help to address chronic depressive features of the illness, as well as preventing manic relapses in the longer-term. Some studies of cognitive behavioural therapy for bipolar disorder have been positive [10-12]. However, a recent large randomised controlled trial found that benefit was limited to a small sub-group of patients [13]. Psychoeducational interventions, which tend to employ some cognitive-behavioural techniques in the context of adjunctive pharmacological management, have emerged as more promising long-term therapeutic options for bipolar disorder [14-18].

Most of the bipolar psychoeducation research to date has focused on group interventions [16-19]. Colom and Vieta have pioneered group psychoeducational interventions for bipolar disorder and have demonstrated that these programmes are effective in preventing relapse, are economically viable and are highly acceptable to patients [16,17,19]. A potential disadvantage of group psychoeducation is the considerable cost in terms of therapist time. As a result, group psychoeducation for bipolar disorder is not routinely available within the National Health Service (NHS). In order to make bipolar psychoeducational material more easily available to patients we have developed a novel, interactive, web-based, psychoeducational package for bipolar disorder which draws heavily on the positive aspects of the Barcelona model [16] and which has been produced in close consultation with patients, their families, carers and health professionals .

To our knowledge, there are no published data on the effectiveness of interactive or web-based psychoeducational programmes for bipolar disorder. The aim of the study is to assess a novel interactive psychoeducational intervention for bipolar disorder within an exploratory randomised controlled trial. The primary objective will be to assess whether those participating in the "Beating Bipolar" programme have improved quality of life compared to those in the control group. Secondary objectives include assessing whether those participating in the Beating Bipolar programme have improved scores on functioning, insight and depressive and manic symptoms and episodes. An assessment of costs and a detailed process evaluation exploring barriers, feasibility and acceptability of the intervention will also be completed. This paper describes the design of the trial.

## Methods/design

This is an exploratory (phase II) individually randomised controlled trial.

### The intervention: "Beating Bipolar"

The intervention was developed using an iterative process involving focus groups with patients, their carers and families and health professionals. The purpose of these groups was to advise on the design and content of the programme, as well as to revise and refine the intervention. Final decisions on the content were made by the research team. This process took 7 months. The primary focus of the intervention is the recognition and self-management of chronic depressive symptoms, depressive relapse and associated functional impairments. The intervention involves a blending of different delivery mechanisms, with initial face-to-face delivery, followed by written and web-based interactive delivery of factual content and ongoing support via a web forum. The web-based content requires the reader to be engaged in a number of interactive exercises in order to maximise long-term retention of the material.

The key areas covered in the package are: i) the accurate diagnosis of bipolar disorder; ii) the causes of bipolar disorder; iii) the role of medication; iv) the role of lifestyle changes; v) relapse prevention and early intervention; vi) psychological approaches; vii) gender-specific considerations and viii) advice for family and carers. The 8 modules are delivered online on a fortnightly basis over a four-month period. In order to maintain engagement, the modules are relatively brief at 20–30 minutes per module. There is an initial face-to-face introductory meeting with participants designed to engage them with the programme as well as to illustrate its use. Thereafter, participants will log onto the website and complete a module. Each module is then discussed within an online forum for participants, which will be moderated by a consultant psychiatrist (DJS). This forum will facilitate peer support and will allow us to provide clarification of module content, answer questions from participants and provide on-going support and engagement. The online forum will also be important in terms of assessing the acceptability of the intervention.

### Outcome measures

The primary outcome measure is the World Health Organisation Quality of Life assessment tool, Brief version (WHOQOL-Bref) [20] (see Table 1). This provides an assessment of overall quality of life. It is comprised of scores within four separate domains (physical health, psychological health, social relationships and environment). It is a reliable, valid and widely-used measure of quality of life in psychiatric out-patient settings [21]. A review concluded that there is currently no quality of life measure for specific use in bipolar patients, however the WHO-QOL is recommended for this population [22].

**Table 1:** Outcome Measures and Assessments

	Baseline assessment	Interim assessment (at end of treatment)	Final assessment (6 mth after treatment)
	By interview at t = 0 months	By post at t = 4 months	By interview at t = 10 months
**Primary Outcome:**			
WHOQOL-Bref	+	+	+

**Secondary Outcomes:**			

MINI assessment	+	---	+

MADRS	+	---	+

YMRS	+	---	+

GAF	+	---	+

FAST	+	---	+

SAI	+	---	+

Use of health service resources	---	---	+

Process measures:			
Interviews Use of software	---	---	+

WHOQOL-Bref: World Health Organisation Quality of Life Assessment, Brief Version; MINI: Mini International Neuropsychiatric Interview; MADRS: Montgomery Asberg Depression Rating Scale; YMRS: Young Mania Rating Scale; GAF: Global Assessment of Functioning Scale; FAST: Functioning Assessment Short Test; SAI: Schedule of Assessment of Insight

Secondary outcomes will examine functioning using the Global Assessment of Functioning (GAF) and Functioning Assessment Short Test (FAST) scales and insight, using the Schedule of Assessment of Insight (SAI). Current depressive symptoms according to the Montgomery Asberg Depression Rating Scale (MADRS) and current manic symptoms according to the Young Mania Rating Scale scale (YMRS), will also be compared between the two groups. Using the Mini International Neuropsychiatric Interview (MINI), the number and severity of depressive and manic symptoms and number and timing of episodes of depression and mania or hypomania experienced during the follow-up period will be compared between groups (this will permit assessments of time to relapse)

### Sample size

Based on our own work and the work of others using the WHOQOL-Bref in mood disordered patients, we expect that the mean score for patients at entry will be 65 (standard deviation 14.4) and that a meaningful clinical response will be a difference between experimental and control groups of 10 or more points [23]. For an 80% chance of detecting this difference on the WHOQOL-Bref, at a significance level of 0.05, 32 patients are needed in each arm of the trial at follow-up. We will recruit 50 patients into each arm of the study which, allowing for a 30% attrition rate, will mean that 35 patients in each arm will complete the trial. Our recruitment strategy and our extensive contacts with local clinical services and patient groups mean that this target of 100 patients recruited into the trial is realistic and achievable.

### Recruitment

Participants will be identified and recruited from multiple sources across South Wales, including Cardiff Primary Care Practices, Cardiff Community Mental Health Teams (CMHTs) and local branches of the Manic Depression Fellowship. Our recruitment strategy will be aided by using the resources of the Mental Health Research Network for Wales (MHRN-Cymru) and the Primary Care Mental Health Research Network in Wales (PCMHRN-Cymru).

### Inclusion criteria

The main inclusion criterion for this study will be a diagnosis of DSM-IV bipolar disorder (including type I and type II) currently in clinical remission. Clinical remission has been chosen as an inclusion criterion because this is an exploratory trial of an intervention which requires participants to be able to fully engage with psychoeducational material. It is also the case that the primary outcome is a measure of quality of life rather than clinical symptoms. Diagnosis will be assessed using the Mini International Neuropsychiatric Interview (MINI) [24] and clinical remission will be defined as not fulfilling diagnostic criteria for a depressive, manic or mixed affective episode during the preceding 3 month period, plus a current Montgomery Asberg Depression Rating Scale (MADRS) score of less than or equal to 10 [25,26] and a Young Mania Rating Scale score of less than or equal to 8 [27]. These MADRS and YMRS threshold scores are widely accepted correlates of symptomatic remission in bipolar disorder. Participants must also be aged between 18 and 65 for inclusion in this study.

### Exclusion criteria

These will include an inability to engage fully in the psychoeducational programme (for example, because of cognitive impairment or not having English language of a sufficient level), not meeting diagnostic criteria for bipolar disorder, and not being in clinical remission according to the definitions above. Given that this is an exploratory trial, no other exclusion criteria will be specified.

### Trial procedures: consent, baseline assessments and randomisation

**Figure 1:**
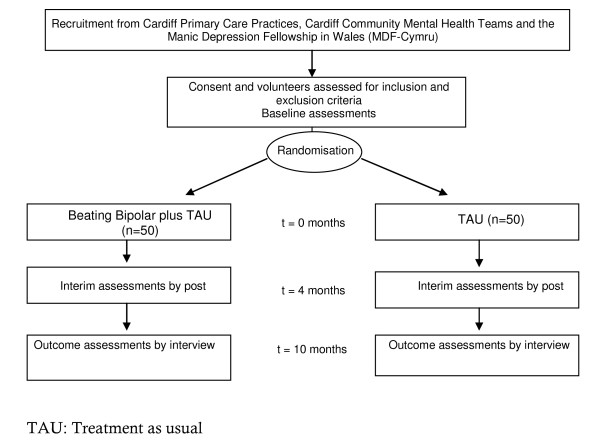
**BIPED Trial Design**.

All potential participants will receive an invitation letter and a detailed information sheet about the study inviting them to approach the research team if they are interested in taking part. They will be asked to provide written consent before being screened for the inclusion and exclusion criteria using the MINI interview and MADRS/YMRS assessments (see Figure 1). Participants identified as eligible will also complete the WHOQOL-Bref [20], the GAF Scale [28], the FAST [29] and the SAI [30]. Participants will be individually randomised remotely using computer-generated number lists to either, the Beating Bipolar intervention plus treatment-as-usual (TAU) or TAU alone.

Delivery of the intervention will begin within 2 weeks of randomisation. Both arms of the study will receive TAU, which will be ongoing care by CMHTs and/or General Practitioners (GPs). We will attempt to minimise potential contamination between groups by giving those in the experimental group individual logins to the website and emphasising the importance of keeping these details private. We will also monitor use of the software and explore possible contamination during the process evaluation interviews. In order to minimise demoralisation and drop-out in the control arm, all participants will be offered the intervention at the end of the follow-up period if the intervention appears to be effective. The intervention will last for 4 months. There will be an interim assessment at the end of the intervention, which will be a postal questionnaire of the WHOQOL-Bref. The primary outcome point will be 10 months from randomisation and all measures will be reassessed at this point (Figure 1 and Table 1).

### Process evaluation

A process evaluation will be conducted to evaluate whether the intervention was carried out in accordance with the trial protocol, to examine the feasibility and efficacy of the programme and to identify barriers to effectiveness as well as areas for improvement. We will map the patients' participation in the forum and use of the psychoeducational programme. We will be able to examine for example, how often participants log in and which pages they use. After the follow-up is complete, qualitative interviews with around 20 of the participants in the intervention group will be conducted to evaluate issues such as acceptability of the intervention, engagement, aspects participants found particularly useful, problems leading to non-adherence and possible contamination between groups. These participants will be purposively sampled to include both males and females from different age groups and will also include a range of levels of engagement with the package.

### Assessment of Costs

We will also conduct an assessment of the direct costs of the intervention and identify its key cost consequences e.g. reductions in demands on general practitioners. Cost of the intervention will be assessed by monitoring resources used and valuing them using standard methods [31]. Participants will be asked to report their use of other NHS resources, social services and time off work. The time they spend on-line will be monitored.

### Analyses

#### Statistical analyses

The primary analysis will be an intention-to-treat analysis and will compare the WHOQOL-Bref scores between the two groups whilst controlling for baseline WHOQOL-Bref scores using ANCOVA. Secondary outcome analyses will be performed similarly controlling for baseline and we will explore the impact of non-engagement with the intervention by undertaking exploratory complier adjusted (CACE) analyses[32]. Analysis of adherence to the program will also be undertaken using logistic regression. No interim analyses or formal subgroups analyses are planned.

#### Qualitative analyses

The interviews will be audio recorded and transcribed. We will employ standard thematic content analysis techniques[33] This method of analysis is essentially a process of summarization, categorisation and counting frequency of responses. The transcripts will be closely examined to identify themes and categories. Codes will be applied to these broad themes which will then be broken down further into sub-codes. Agreement on concepts and coding will be sought between members of the research team in order to ensure reliability. We will seek to identify commonly expressed themes as well as unusual cases. A proportion of the data (20%) will be coded by two different team members to check on reliability of the coding scheme. The interviewing will be iterative; where new themes emerge we will incorporate them into the interviews.

#### Analyses of costs

As this is an exploratory study a full economic evaluation would be premature. Nevertheless, the assessment of the costs and cost consequences discussed above will inform the design of the definitive study (if justified by the results of this exploratory study) by identifying the key cost drivers. For example, if the intervention is shown to have a major impact on productivity (assessed by monitoring time off work) then an NHS perspective would not be appropriate for the economic evaluation to be undertaken alongside the definitive trial. The exploratory trial will only assess costs, since for cost effectiveness purposes a utility based generic measure of health status such as the EuroQol (EQ-5D) [34] would be needed to produce an incremental cost effectiveness ratio which could allow comparison with other interventions. This is not necessary at this stage.

## Discussion

This trial will be the first to evaluate the effectiveness and acceptability of a novel web-based psychoeducational intervention for bipolar disorder. This exploratory trial will assess the impact of the programme on the quality of life, functioning and symptom profile of patients with bipolar disorder. In combination with the economic evaluation and the process measures assessing acceptability and engagement with the intervention, data from this trial will substantially inform the design of a larger (phase III) randomised controlled trial of this intervention.

### Strengths of the study

We have utilised our experience in the development and evaluation of web-based learning programmes in different topic areas to maximise the design aspects and usability of the programme[35]. In addition, we have involved patients, their families, carers and health professionals in the development of the content and design. We have also incorporated findings from recent studies into the design and content of the intervention. For example, the content draws heavily on the positive aspects of a group based psychoeducation programme which has been successfully evaluated in a trial[16]. A recent qualitative study examining patients views of a group based psychoeducation intervention identified three main themes of importance; the treatment of bipolar disorder, perception of others and the support of the group [36]. These aspects are all included in this programme.

We have included a module specifically for family or carers of bipolar patients because we felt that their support and guidance was instrumental in the way that bipolar patients cope with their illness. One recent study examining a psychoeducation intervention for caregivers of bipolar patients in remission found a reduction in mood recurrence and longer intervals without relapse for manic or hypomanic episodes[37].

### Challenges

In order to ensure that the programme would be available to as many participants from different backgrounds as possible and to allow those who do not have internet access at home to be involved in this study, we will liaise with local libraries in South Wales to provide free internet access and support for those participants who require it.

Encouraging patients with a bipolar diagnosis to engage with the programme may be challenging given the cognitive deficits and emotional difficulties associated with the condition. However, we will be recruiting patients who are in remission and we have tried to design the programme to take account of these issues. We have paid close attention to engagement, from the initial meeting through the general appeal and interactivity of the programme to the peer support via the forum. One of the factors affecting the success of group psychoeducation programmes is likely to be peer group support. We have tried to incorporate this via the initial meeting and the forum. However it remains to be seen whether the forum peer support and feedback can sufficiently fulfil this role.

It has been suggested that psychoeducation should be delivered as part of a routine package of care for bipolar disorder. A systematic review concluded that there was evidence that psychoeducation enhances patients' knowledge of the disorder and of treatments available. It also leads to decreases in relapse and improves compliance with treatment[38]. The authors recommend psychoeducation in the management of bipolar disorder. This web based method of delivering psychoeducation could provide a cost-effective method for achieving this.

## Conclusion

In summary, bipolar disorder is common, under-recognised and poorly managed. It is associated with considerable personal and social impairment and as a chronic, life-long and relapsing condition it has an enormous impact on the economy. As noted above, there is an urgent need to develop treatments which are simple, efficacious, cost-effective, acceptable to patients and that have the potential to be widely applied. This trial will assess whether Beating Bipolar fulfils these criteria.

## Competing interests

The authors declare that they have no competing interests.

## Authors' contributions

SS drafted this paper which was added to and modified by all other authors. DJS, IJ and SS wrote the content of the Beating Bipolar package. EB and DJS conducted the focus groups. SS, DJS, EB, EG, IJ, KH, DC and NC contributed to the design of the study protocol. All authors read and approved the final manuscript.

## Pre-publication history

The pre-publication history for this paper can be accessed here:


